# Management in Fallot Tetralogy associated with Congenital Scoliosis Case Report

**Published:** 2012

**Authors:** Stanev Ximena

**Affiliations:** “Carol Davila” University of Medicine and Pharmacy, Obstetrics and Gynecology Department, “Malaxa” Clinical Hospital, Bucharest, Romania

**Keywords:** congenital heart malformation, congenital scoliosis, treatment, Ao = aorta, PA = pulmonary artery, PAP = pulmonary artery pressure, RV = right ventricle, RVP = right ventricular pressure, RVH = right ventricular hypertrophy, RBL = right block line, LV = left ventricle, VSD = ventricular septal defect, SIA = interatrial septum, SIV = interventricular septum, BP = blood pressure, BPs = systolic blood pressure, CT = computed tomography, VEPTR = vertebral expandable prosthetic titanium rib

## Abstract

Tetralogy of Fallot is the most common congenital heart malformation that produces cyanosis. It consists of four different defects of the heart: ventricular septal defect, pulmonary artery stenosis (blockage of blood flow from the right ventricle to the lungs), right ventricle hypertrophy and dextroposition of aorta. Echocardiography is essential in establishing the diagnosis of patients with cardiac malformation. Patients with Fallot tetralogy present a higher frequency of major non-cardiac congenital disorders. The association with congenital scoliosis influences vital and functional overcomes, restricting physical activity and lowering life expectancy. The author presents therapeutic management on the clinical case of a 13-year-old child with Fallot tetralogy and congenital scoliosis. Therapeutic management of both illnesses consisted in serial surgical interventions as it follows: first time at the age of six years, cardiac malformation was solved, and later, at the age of 13 years the correction of spinal deformity was performed.

## General information

Tetralogy of Fallot is rare, but it is the most common form of congenital cyanogen heart disease. The severity of symptoms depends on the size of ventricular septal defect and pulmonary stenosis degree.

If the **stenosis is mild**, the pressure in the right ventricle can be slightly higher than that of the left ventricle, so that a small amount of (neoxygenated) unoxigenated blood from the right ventricle will pass into the left one, mixing with oxygenated blood. These children will have lower levels of oxygen blood, but they **are not cyanotic**.

**A more severe obstruction** of the pulmonary artery will cause the passing of a bigger amount of unoxygenated blood through the septal defect in the left ventricle, mixing it with oxygenated blood. This will be sent to the systemic circulation and will lead to cyanosis.

Tetralogy of Fallot is rare, but it is the most common form of congenital cyanogen heart disease. The factors raising this risk condition along prenatal period consist of alcoholic mother, diabetes mellitus, mother aged over 40, deficient nutrition, rubella and other viral diseases. There is a higher incidence of chromosomal abnormalities (such as Down and DiGeorge syndromes) in children with Fallot tetralogy [**11**].

**Four diagnostic subgroups of Fallot tetralogy** are described:

a. Tetralogy of Fallot without pulmonary valve syndrome: pulmonary valve is dysplastic and pulmonary arteries are dilated, often associated with difficulties of breathing;

b. Tetralogy of Fallot with common atrioventricular canal;

c.Tetralogy of Fallot with pulmonary atresia associated with hypoplasia of pulmonary arteries;

d.Tetralogy of Fallot with pulmonary stenosis, the most common form, where stenosis can be supravalvular, valvular, subvalvular or combined.

Definitive treatment of tetralogy of Fallot consists of a surgical correction. Timing of surgery remains controversial but most agree that the presence of severe cyanosis or hypercyanotic spells need a surgical intervention. A complete repair consists in closing the ventricular septal defect with a patch and enlarging the right ventricular outflow tract. The latter usually requires the incision across the pulmonary valve annulus and the placement of a patch of synthetic material to widen the outflow tract at all levels of obstruction [**4,5,8,10**].

When surgical intervention is necessary in a patient who is not a good candidate for a complete repair (i.e., very small patient size, tiny pulmonary arteries or an anomalous coronary artery course), a palliative procedure is performed. Palliation consists of the placement of a shunt from the aorta in the pulmonary artery to increase pulmonary blood flow. The most commonly performed shunt today is the modified Blalock-Taussig shunt, in which a tube of Gore-Tex is placed between the subclavian artery and the pulmonary artery [**1**].

**Patients** with **tetralogy of Fallot** have a higher incidence of major non-cardiac defects (i.e., **scoliosis congenital** with neurological abnormalities).

**Congenital scoliosis** consists of congenital vertebral anomalies caused by defects of forming, segmentation, or both (combined).

The congenital vertebral anomalies can be balanced or unbalanced, causing a severe deformity of the spine, which can evolve in advanced stages to cardio-respiratory failure and / or neurological damage. The evolution of untreated congenital scoliosis (McMaster & Ohtsuka, 251 patients, 1982) may lead to (can determined in 14% and respectively in 75% of cases) mild deformities in 14% of the cases and to major deformities in 75% of cases [**9**]. In 35% of the cases, congenital scoliosis is associated with neurological abnormalities (diastematomyelia, Chiari malformation, intradural lipoma, myelomeningocele), 25% with congenital heart diseases (DSA / DSV, tetralogy of Fallot, transposition of great arteries), 20% with genitourinary defects (horseshoe kidney, renal aplasia, duplicate ureter, hypospadias). Diagnosis is done on imagistic basis: CT, 3D- CT, MRI [**2**].

The therapy of congenital scoliosis is surgical and consists of the following:

1. In situ fusion
2. Convex side hemi-epiphysiodesis (partial growth arrest)
3. Somatic instrumentation following resection of haemivertebra and discectomy
4. Spinal osteotomy
5. Segmental spinal resection (2-5 vertebrae)
6. VEPTR implantation (Vertebral Expandable Prosthetic Titanium Rib ) [**9**]

Haemivertebra excision allows the correction of the spinal deformity and trunk imbalance. The technique consists of a somatic transpedicular instrumentation using both anterior and posterior surgical approaches, or simply a posterior instrumentation [**2**].

VEPTR is indicated for thoracic insufficiency syndrome (TIS) (Campbell 1993) caused byspatial deformity of thorax [**3**].

## Case presentation

**Fig. 1:**
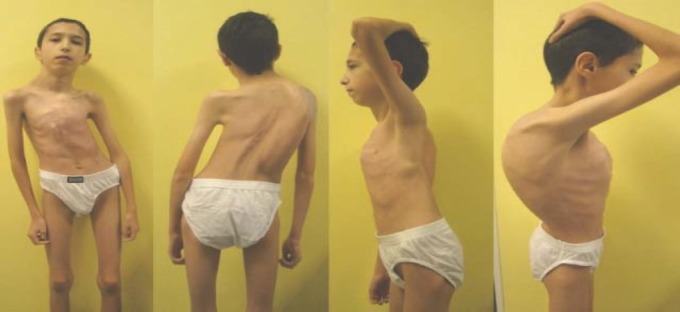
Clinical relevant aspects for the anterior chest wall deformation, the spine deformation (the right thoracic curve) and gibbous rib deformity

Male child, aged 13 years, known with congenital cyanogen heart malformation (tetralogy of Fallot) surgically treated at the age of 6 years, admitted for worsening respiratory dysfunction secondary to severe medical neglected scoliotic deformities. The child had dyspnea, palpitations and fatigue with moderate exercise, without signs of congestive heart failure. Cardiac symptoms imposed the necessity of a sustained medical treatment with angiotensin converting enzyme inhibitors (ACE-captopril and Tritace) and postoperative periodic cardiological controls, until the present. **Family history record** did not point the existence of a family history of congenital heart disease, the child having a healthy twin. From the **personal physiological data**, what had to be remembered was the fact that he was a premature baby, resulted from twins, with weight at birth 1700g, APGAR score = 8. The **medical history** includes **right inguinal hernia** operated at the age of 2 months, **neglected hypospadias, cyanogen congenital heart malformation (tetralogy of Fallot)**, treated surgically at the age of 6 years when the child was able to live an active life with acceptable tolerance to moderate efforts. **Clinical examination** reveals cold and pale extremities with discreet perioral cyanosis, in a child with staturoponderal deficit. The precordial region is deformed with asymetric pectus carinatum, more convex on the right side and a smooth surgical scar is visible on the middle part of the sternum. The apexian shock is present in the 5^th^ intercostal space on the middle-clavicular line, pulse rate = 100/min, BP = 100/60 mm Hg, rhythmic heart beats, a systolic rough murmur may be heard with maximum intensity in the focus of pulmonary with large area of presternal irradiation. The peripheral pulse is present bilaterally without clinical signs of congestive heart failure. O2 systemic saturation determined by pulse oximeter is SpO2 = 96%. At inspection of the spine, the deviation of the axis of the thoracolumbar spine is noticed in the frontal plane, with a right convex thoracic curve, which is not corrected by lateral bending, the right side gibbous costal deformity pronounced by movements of flexion of the spine, asymmetry of the shoulders with higher right scapula and asymmetry of the pelvis with apparent inequality of limb (**Fig. 1**) without limb neurological disorders. Abdominal palpation reveals an enlarged liver, with the inferior edge at 2.5 cm below the rib angle.

On **laboratory findings,** blood tests and liver function tests were within normal limits. **Spirometry** shows restrictive moderate respiratory failure.

**Electrolytes** and the **partial pressure** of **O2** and **CO2** levels were normal.

**Preoperative transthoracic echocardiography performed at the age of 6 years,** showed Ao = 1.7 cm, Ao asc = 1.8 cm, aortic valve opening = 1.04 cm, RV = 2.4 cm. Posterior mitral valve with reduced mobility. Tricuspid regurgitation grade I / II. Pulmonary flow: V max = 5.7 m / s, Pmax = 132 mmHg, Pmed = 73 mmHg. Pulmonary artery diameter ring AP = 0.9 cm, pulmonary artery trunk=2.5-2.6 cm. Valvular and infundibular pulmonary stenosis. Severe right ventricular hypertrophy (RVH) and important enlargement of right ventricle. Ventricular septal defect (VSD), subaortic approximately 0.3 cm with right-left shunt, Vmax = 4.4 m / s, Pmax = 78 mmHg, interatrial septum (SIA) apparently intact. Dilated coronary sinus, throbbing.

**Conclusion:** Valvular and subvalvular pulmonary stenosis. Ventricular septal defect (VSD) with right-left shunt. Dilated right cavities. Right ventricular hypertrophy (RVH). Aortic dextroposition. Suspected mitral valve modified.

**Fig. 2:**
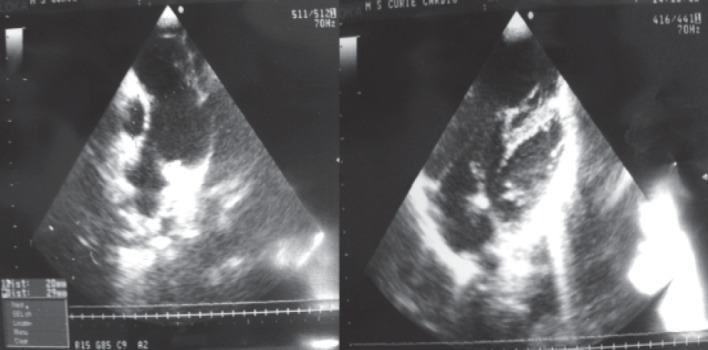
Transthoracic ultrasound examination after surgery, at the age of 13 years, showing dilated right heart, requiring repeating echocardiography exam after 6 months

**Transthoracic echocardiography** (**Fig. 2**) performed **after surgery at the age of 13 years,** identified the right heart as being much larger than the left heart (which looked slightly hypoplastic). The apex was not formed by left ventricle.

LV volume = 21 ml, RV volume = 85 ml, peak-apex diameter LV = 12 mm, peak-apex diameter RV = 25 mm. Without residual lesions after surgery. SIV intact, Ao ring = 20 mm, PA = 29 mm, dilated. Tricuspid regurgitation gr. II-III. PAPs = 40 mm Hg.

**Fig. 3:**
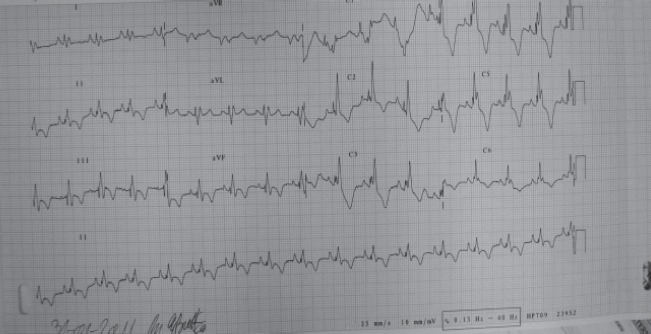
ECG appearance

**ECG** showed sinus rhythm, heart rate frequency 90/min, QRS axis deviated to right, right ventricular hypertrophy (when R wave was overriding in V1-V2), QRS complex was widened, with bifid, notched R wave, having the appearance of right block line (RBL) and subendocardial ischemia (symmetrical, sharp, negative T-waves in V1-V6, DII, DIII, aVF) **(Fig. 3)**.

**Fig. 4:**
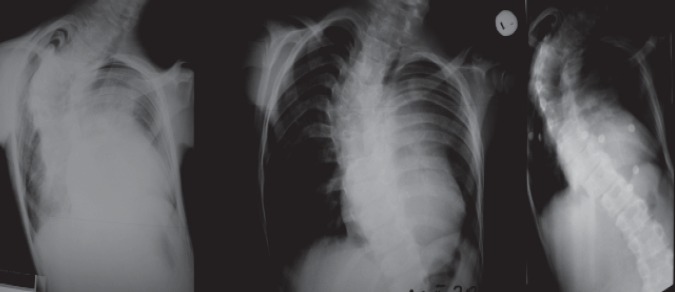
Preoperative radiological examination at age 13

**Chest X-rays** showed asymmetric thorax with left hypoplasia, cardiomegalia with enlargement of cardiac silhouette, dilated pulmonary arch, aortic arch dextroposition over the right ventricle (deviation of the aortic origin to the right), decreased pulmonary circulation in the periphery (**Fig. 4**).

**Spine X-rays** indicated thoracolumbar scoliosis, with right convex thoracic curve.

### Spine Computed Tomography (CT)

**Fig. 5:**
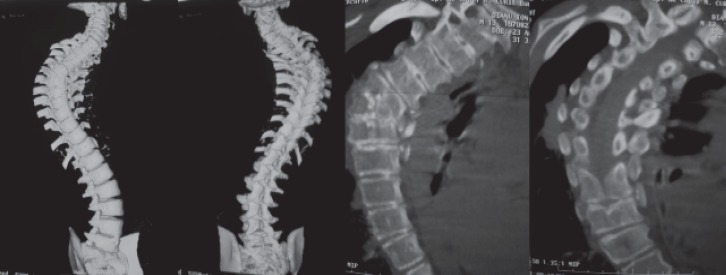
Thoracolumbar congenital scoliosis with hemivertebra T5 at right side

"S" thoracolumbar scoliosis with convex thoracic right curve with peak at T5 and the lumbar curve peak at L4 (**Fig. 5**).

Right T5 hemivertebra, with lack of identification of the left vertebral pedicle, but with the corresponding rib arch.

Based on general clinical examination, the morpho-functional exploration, laboratory and imaging, the child was **diagnosed** with:

**1. Congenital thoracolumbar scoliosis with right thoracic curve and non segmented hemivertebra T5-T6 on the right side**

**2. Tetralogy of Fallot**

a. Interventricular septal defect
b. Presence of foramen ovale
c. Severe stenosis of the pulmonary artery at infundibular and valvular side
d. Severe hypertrophy of the right ventricle and interventricular septum
e. Suprastenotic aneurismal enlargement of the pulmonary artery trunk

**3. Left lung hypoplasia**

**4. Abnormal origin of the left pulmonary artery from the crossing right pulmonary artery** and compressed tracheo-bronchial fork.

**5. Superior vena cava with drainage in coronary sinus**

**6. Supranumerary bronchia with origin in trachea for the upper lobe of the right lung (bronchus suis) Treatment** was conducted in two stages:

**1. Treatment of the heart disease (tetralogy of Fallot)** at the age of 6 years

**2. Treatment of the congenital scoliosis** at the age of 13 years

### 1. Treatment of the heart disease (tetralogy of Fallot)

**The surgical indication** was established based on the clinical exam performed by the cardiovascular surgeon (cyanosis, fatigue at medium/ small intensity exercise) and through morpho-functional exploration (transthoracic echocardiography showing four heart defects suggestive for tetralogy of Fallot).

**Surgical treatement** consisted in a fully correction of the congenital heart malformation, aiming the interventricular septal defect closure with patch, pulmonary infundibular and right ventricle modeling resection; plastic expansion of pulmonary valve ring with autologous pericardium patch fixed in glutaraldehyde. The intervention was conducted by using extracorporeal blood circulation.

**Intraoperatively,** there was significant hypertrophy of the walls and pillars of the right ventricle as well as supravalvular dilation of the pulmonary artery. The measurement of blood pressure (BP) = 87/49 mmHg (64 mmHg), right ventricular pressure (RVP) = 52/26 mmHg (37 mmHg) and pulmonary artery pressure (PAP) = 39/24 mmHg (30 mmHg).

**Immediate postoperative evolution** was difficult, with low cardiac output syndrome, central hyperthermia and peripheral circulatory failure (cold extremities).

Among **postoperative complications** the following were included:

1. Low cardiac output syndrome (increased requirement of tonicardiac drugs)
2. Gastrointestinal bleeding externalized with hemathemesis and melena with severe post hemorrhagic anemia controlled by the administration of electrolytes and hematological rebalancing therapy
3. Massive pneumothorax, first requiring aspiration, reappearing in evolution, later being treated conservatively
4. Sepsis with positive blood cultures for E. coli, controlled therapeutically

**At discharge,** the child was afebrile, hemodynamically stable and breathing, with sinus rhythm and surgical wounds healed. X-ray exam thoracic performed before discharge showed thin air blade in resolution from previous exams.

### 2. Treatment of congenital scoliosis

At the age of 13 years, worsening restrictive ventilatory dysfunction secondary to spinal deformity required an urgent decision of the surgical treatment, which was carried out sequentially in two stages through double approach (anterior and posterior) of the unsegmentated supernumerary hemivertebra T5-T6 by right side.

The treatment consisted in hemiarch-hemivertebra excision and discectomy T3-T8 by postero-lateral thoracic approach through the right extrapleural space, somatic transpedicular rachisynthesis T4-T7, thoracoplasty with posterior rib arches excision C3-C8 and rachisynthesis with posterior instrumentation after 7 days.

Excision of hemivertebra allowed spinal deformity correction, realizing the desire of obtaining a "spine without imbalance of shoulders and pelvis and without neurological deficit." [**2**].

The surgical treatment was very difficult, powerful complications as **myelitis** (paralysis, paraplegia, incontinence), **visceral** (lung, heart), **vascular** (thoracic duct injury & large vessels), **muscle, urogenital** and **peripheral nerve injuries** being possible [**7**].

Initially, in order to avoid these complications, the application of a VEPTR device (Vertical Expandable Prosthetic Titanium Rib) for thoracic expansion and correction of scoliosis was proposed, but the acquisition of the system was not possible.

## Discussion

**Resection hemivertebra** and **somatic instrumentation** of several over-and underlying vertebrae avoids increasingly marked deformity in evolution.

Somatic rachisynthesis associated with discectomy and hemivertebra resection through double approach allowed curves correction and maintaining scoliosis long term correction [**2**].

**VEPTR devices** (Fritz Hefti) are an alternative treatment for thoracic insufficiency syndrome (TIS) in cases of severe thoracic deformities associated with scoliosis [**6**].

They are designed to mechanically stabilize and enlarge thorax, for improved breathing and for a better development of the lungs, for the prevention and correction of scoliosis, being successfully used in congenital scoliosis and idiopathic infant scoliosis [**2**].
